# Association between Stress at Work and Temporomandibular Disorders: A Systematic Review

**DOI:** 10.1155/2021/2055513

**Published:** 2021-05-15

**Authors:** Ricardo Luiz de Barreto Aranha, Renata de Castro Martins, Diego Rodrigues de Aguilar, Johana Alejandra Moreno-Drada, Woosung Sohn, Carolina de Castro Martins, Mauro Henrique Nogueira Guimarães de Abreu

**Affiliations:** ^1^School of Dentistry, Universidade Federal de Minas Gerais, Belo Horizonte, Minas Gerais, Brazil; ^2^Department of Community and Preventive Dentistry, School of Dentistry, Universidade Federal de Minas Gerais, Belo Horizonte, Minas Gerais, Brazil; ^3^Department of Population Oral Health, School of Medicine, The University of Sydney, Sydney, New South Wales, Australia; ^4^Department of Child and Adolescent Health, School of Dentistry, Universidade Federal de Minas Gerais, Belo Horizonte, Minas Gerais, Brazil

## Abstract

Temporomandibular disorders (TMD) have been traditionally associated with psychosocial factors; however, occupational stress as a factor related to TMD has not been adequately assessed in the literature. The aim was to investigate the association between stress at work and TMD on adult paid workers. An electronic search included PubMed, Scopus, Web of Science, Embase, and LILACS databases. Manual searches in the included articles' reference and gray literature were performed. There were no restrictions regarding language or publication period. The inclusion criteria comprised observational studies with paid workers of any category, of both sexes, above 18 years old, assessing occupational stress/stress or distress and TMD as diagnosis or isolated signs and symptoms. Methodological quality was evaluated using Joanna Briggs tools. We narratively assessed the evidence using the Grading of Recommendations, Assessment, Development, and Evaluation (GRADE) approach. We collected 12 studies. 50% reported a positive association between stress and TMD diagnostic across various job categories. On the other hand, TMJ sounds (a TMD sign) and work stress were associated only in a musicians' population. However, the shortage of eligible articles and the methodological limitations provided a very low certainty of the evidence; only 4 of the studies used validated tools for both stress and TMD (2 reporting positive association). The association between stress and TMD is inconclusive by the available data. In the future, we expect more robust epidemiologic studies addressing these relevant aspects.

## 1. Introduction

Temporomandibular disorder (TMD) is a condition of pain or musculoskeletal dysfunction that affects the face in its masticatory structures and encompasses a group of changes involving the temporomandibular joints. It represents the primary cause of nondental pain in the orofacial region [1], and it is the most prevalent chronic pain [1, 2]. Like chronic pain in general, TMD is defined as a clinical and public health problem [3]. Due to extensive variations in the methodological criteria employed, there is considerable variation in the prevalence of TMD signs and symptoms in epidemiological studies (from 3% to 80%) [4, 5]. The TMD diagnostic concepts represent a matter of debate over the past decades, evolving from sparse TMD signs and symptoms to the well-structured Research Diagnostic Criteria for Temporomandibular Disorders (RDC/TMD). Its upgraded version is the Diagnostic Criteria for Temporomandibular Disorders (DC/TMD) Consortium Network, a worldwide effort to improve and standardize the diagnostic tools for research and clinical purposes [6]. Despite the historical lack of robust diagnostic standards, TMD is considered more frequent in adults or young adults, between 20 and 50 years old [1, 4] and in women compared to men (from 2 to 3 : 1) [7–10].

Work environment and work conditions are historically known as disease-related factors, particularly in the face of job instabilities and the high level of performance demands that characterize the current globalized market. The fast changes in technology and local economic conditions present new challenges to work human resources worldwide. Accordingly, paid work involves several situations and aspects that interact with the social determinants of health [11].

The relationship between TMD and stress is well established and widely explored in the literature [12–14]. However, the connection between work factors or work stress in TMD is not sufficiently investigated and, hence, poorly understood and determined. Work stress is a category of psychological stress, defined as a process in which the individual perceives work demands as stressors, which, when exceeding their coping skills, provoke adverse reactions in the subject [15]. High levels of demands, lack of resources, social support [16–18], and low psychological detachment from work [19] stand out as work risk stressors. On the other hand, anxiety is the anticipation of future threats; it is distinguished from fear, the emotional response to a real or perceived imminent threat [20, 21]. The distinction between stress and anxiety is subtle. Both are emotional responses with similar coping mechanisms, but an external trigger typically causes stress. Anxiety is defined as persistent worries, even in the absence of an objective stressor [22]. The limited number of studies dedicated to occupational stress and TMD frequently dealt with physical aspects of work, particularly those directly affecting the orofacial region [23–25]. The TMD field has shifted from etiological and therapeutic mechanical centered to a broader biopsychological disease model, including medical, social, and psychological variables. This change implies that relevant demographics and socioeconomic factors should be taken into account in current research efforts [26]. The psychological literature still reports the term “distress” (a particular categorization of stress, in opposition to “eustress”) as a negative counterpart, the most known type. Distress is the aversive, negative state in which coping and adaptation processes fail to return an organism to physiological or psychological homeostasis [27, 28]. Moreover, the correct identification of etiologic factors will enable the appropriate and comprehensive dental care planning for TMD. This review evaluates the scientific evidence on the relationship between stress/distress/work stress and TMD. Hence, the aim of this study was to investigate the association between stress at work and TMD on adult paid workers.

## 2. Materials and Methods

This systematic review followed the Preferred Reporting Items for Systematic Reviews and Meta-Analyses (PRISMA) checklist [29]. The review protocol is registered at the International Prospective Register of Systematic Reviews (PROSPERO) under the registration number #CRD42020186274.

The review question was as follows: Is there an association between stress at work and temporomandibular disorder among adult workers?

The question mirrors the following PECO framework for observational study development:

Population (P): professional or semiprofessional (part-time) adult workers.

Exposure (E): high levels of stress/distress or stress at work.

Comparator (C): no stress/distress/stress at work or lower stress/distress/stress level at work.

Outcomes (O): TMD or isolated signs/symptoms of TMD.

### 2.1. Eligibility Criteria

The inclusion criteria were as follows: observational studies (cross-sectional, case-control, and cohort studies) evaluating the association between occupational stress, stress or distress among job/work/profession groups and TMD categories, or TMD signs and symptoms; assessing paid professional or semiprofessional (part-time) workers of any type or geographic location, from both sexes and above 18 years old. There was no limit on language and period. The search was updated until March 19, 2021. If any manuscripts written in languages other than English, Spanish, or Portuguese were identified, proofreading would be accessed for a professional translation.

The exclusion criteria were studies assessing nonpaid workers, studies without TMD measures or their signs and symptoms, surveys that assess other psychological disorders ruling out stress/distress, or disallowing the analysis of the association between the variables.

### 2.2. Search Strategy

We searched the following electronic databases from inception up to September 2020: Medline through PubMed, Scopus, Web of Science, and Embase through Ovid, and Latin American and Caribbean Health Sciences (LILACS) through the Virtual Health Library (BIREME). We also searched gray literature through OpenGrey and Google Scholar; these latter limited to the first 100 listed results. We hand searched the list of references of included studies. Details of the search strategies are listed in supplementary file 1.

### 2.3. Study Selection and Data Extraction

The list of references was retrieved from Endnote web (http://myendnoteweb.com) (Clarivate Analytics, PA, USA). Two independent examiners screened titles and abstracts and selected papers in the forthcoming stages (Cohen's Kappa = 0.937). Titles and abstracts that met the eligibility criteria were selected for full-text analysis. A second screening was independently performed based on the full texts. A third examiner was consulted to solve any eventual disagreement.

A spreadsheet was created at the Excel program for data extraction (supplementary file 2). The independent reviewers tested the form. Data regarding the name of the authors, date of publication, study settings, and population characteristics (country, sample size, dropouts, control group, occupational stress/stress reports, diagnosis of TMD disorders, modified or impaired mandibular movement, and TMD joint pain or joint sounds registered as TMD signs/symptoms) were collected. The assessment of muscle pain and joint disorder was performed according to the Diagnostic Criteria for Temporomandibular Disorders (DC/TMD).

### 2.4. Quality Assessment of Original Articles

Joanna Briggs Institute's tools for cross-sectional studies were used to assess the methodological quality [30].

For the included cross-sectional studies, the following criteria were considered: inclusion criteria, study subjects, exposure measures, objective and standard criteria, confounding factors and strategies to deal with confounding factors, outcomes measures, and appropriate statistical analysis.

For electing the essential confounding variables, we consulted the DC/TMD [6] and the heuristic model of “The Orofacial Pain: Prospective Evaluation and Risk Assessment (OPPERA).” The latter is a multicenter ongoing cohort study from a large base of TMD-free adults, assessed in detail several years for phenotypic and genetic predictor factors of first-onset TMD [9]. For the stress domain, similar confounding factors were considered [31–36]. In the end, the minimum appropriate confounding factors selected to integrate the adjusted analysis were anxiety, depression, gender, age, sleep disturbances, headaches, and comorbid systemic diseases related to pain (e.g., diabetes, fibromyalgia, or rheumatoid arthritis).

### 2.5. Data Synthesis

For the final narrative synthesis, we used the Grading of Recommendations, Assessment, Development, and Evaluation (GRADE) to assess the certainty of the evidence for narrative synthesis [37]. For observational studies, the certainty of the evidence starts with low, and it can be rated down due to risk of bias, inconsistency, indirectness, imprecision, and publication bias. The evidence was further assessed for dose-response, the effect's magnitude, and residual confounders that could rate up the certainty [38].

## 3. Results

**Figure 1:**
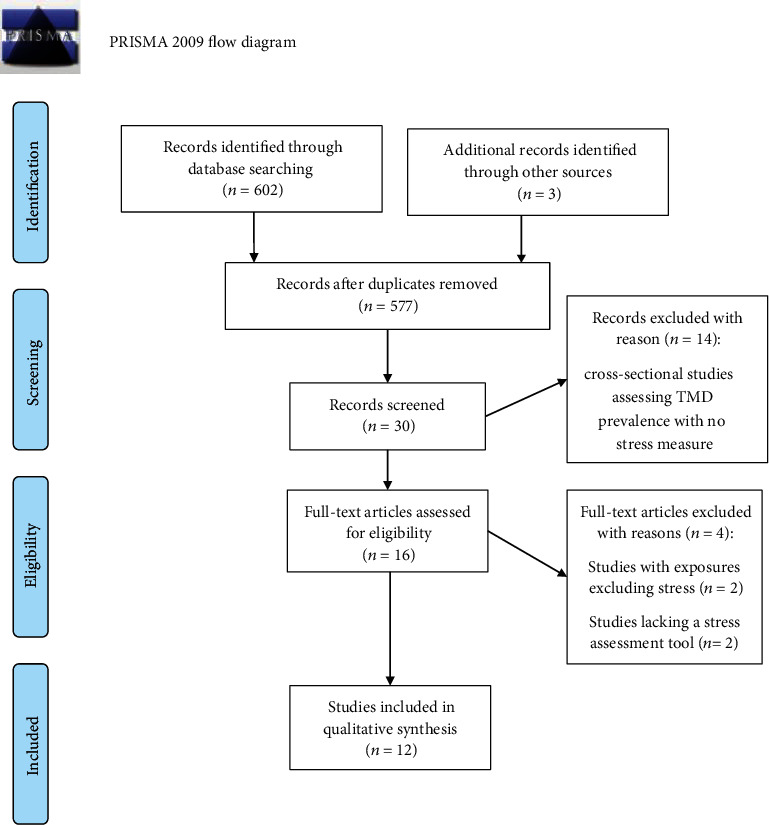
Flowchart showing the criteria of article search and selection (adapted from PRISMA).

The initial search retrieved 602 studies. The search in the reference lists of articles and gray literature provided three additional items. After removing duplicates and the first screening of titles and abstracts, 577 articles remained. Thirty studies were full-text analyzed. Fourteen were excluded because they were cross-sectional inquiries for assessing TMD prevalence in professional categories without reporting stress. Four studies were excluded because they did not allow an association analysis between TMD/signs/symptoms and stress. Finally, 12 studies were included in the systematic review (Figure 1) [39–50].

### 3.1. Quality Assessment of Original Articles

The implementation of Joanna Briggs Institute's tools for cross-sectional studies yielded for each domain investigated. From the eight criteria evaluated, that with the highest adherence was about objective, standard criteria for measurements (item 4) and that with the lowest adherence to JBI evaluation was about confounding factor identification (item 5) (supplementary file 3).

### 3.2. Narrative Synthesis

Due to the significant heterogeneity among studies, different types of workers assessed, various diagnosis tools for DTM/stress, and distinct statistical methods, a narrative synthesis was performed instead of a meta-analysis to evaluate the association between stress at work and TMD. From the 12 studies, 11 presented the diagnosis of TMD [39–44, 46–50], and 1 evaluated only the signs and symptoms of TMD [45]. Two manuscripts evaluated both diagnosis and signs and symptoms of TMD (Table 1) [41, 50]. To perform metaregression, at least a sufficient number of studies in the model are necessary [51]. Few studies informed about participants with or without TMJ (*n* = 5), and other few informed about low and high levels of stress (*n* = 5). None had similar work class categories. Therefore, a metaregression analysis with regard to stress and TMJ was not feasible too.

The narrative synthesis showed that six studies found a positive association between stress and the diagnosis of TMD [39, 40, 43, 46, 47, 49]. The highest association strength was an OR = 6.03, 95% CI 2.51–15.33 [43]. However, among these studies, only 2 used validated scales for stress [43, 49], 3 used nonvalid scales [40, 46, 47], and one study [39] was not clear as the scale used for stress. Concerning the TMD, 4 used validated scales [40, 43, 47, 49], and 2 did not [39, 46]. The work categories varied from dentists, high-tech workers [40], employees of Finnish Broadcasting Company [39], Asian military personnel [49], full-time female workers [47], information technology professionals [46], and violinists (Table 1) [43].

On the other hand, five articles reported no association between stress and the diagnosis of TMD [41, 42, 44, 48, 50]—2 studies using a validated scale for stress [44, 48] and 3 using nonvalid scales [41, 42, 50]. All of them used TMD valid instruments. The work categories varied from vocalists [41], upper strings instrumentalists (violin, viola) and wind instrumentalists [50], nurses [48], industrial workers [44], and electronic industry workers [42] (Table 1).

For the studies with TMD signs and symptoms, one found an association with stress (for joint sounds) [50], and two did not [41, 45]. All used an original validated TMD scale but nonvalid stress scales. The work categories varied from vocalists [41], upper strings instrumentalists and wind instrumentalists [50], and workers from call centers [45] (Table 1).

The use of validated scales has provided a different associative rate among studies. From the twelve evaluated manuscripts, only four articles employed validated tools for both variables stress and TMD. From this subgroup, 2 found an association [43, 49], and 2 did not find it [44, 48].

In summary, from the 12 articles, 7 found an association between TMD diagnostic/signs and symptoms and stress [39, 40, 43, 46, 47, 49, 50] and 5 did not [41, 42, 44, 48, 50].

The evidence's certainty was very low (Table 2), rated down due to the risk of bias, inconsistency, indirectness, and publication bias. There were very serious problems of risk of bias. All studies did not adjust for the selected confounders. Seven out of 12 papers used nonvalidated scales for stress [40–42, 45–47, 50] and 2 used nonvalid scales for TMD [39, 46], which means not using a validated method to measure the exposure or the outcome. There were very serious problems due to indirectness as the evidence is from some types of workers, with limited applicability to all workers. The majority of the evidence was from studies that evaluated stress at work. Only two studies assessed occupational stress with specific questionnaires [43, 48]. The certainty of the evidence was rated down due to publication bias. According to the GRADE approach, publication bias is strongly suspected for observational studies as registries are nonmandatory [52].

## 4. Discussion

Despite mostly manuscripts found an association between work stress and TMD, there was very low certainty about this association (below the original low certainty stipulated for observational studies). Further, there were severe problems of risk of bias. Hence, there is a combination of lack of association, inconsistencies in outcome and exposure, nonstandardized scales, and low quality of the evidence in the observational studies that evaluated the association between work stress and TMD.

There was heterogeneity among included studies and instruments to measure the outcome and the exposure. The high discrepancy of association results found over articles points to a high degree of inconsistency. Differences in the diagnosis criteria and the exposure could result in different findings, and this issue has been comprehensively discussed on other healthcare issues [53, 54]. The variation of TMD diagnostic criteria may impact its prevalence [1], and in our systematic review, it has probably changed the rate of association between stress and TMD. Anamnesis is the essence and starting point for any TMD diagnosis, represented by functional questionnaires in the research setting, whether alone or within the entire RDC/DC TMD framework, including clinical, imaging, or laboratory exams, depending on the case. Most TMD functional questionnaires applied in epidemiological surveys over time have addressed a TMD diagnostic concept that does not differ between articular and muscular TMD or yet painful and painless conditions [55–57]. Accordingly, eventual articles employing instruments for assessing specific diagnoses like painless TMD symptoms [39], myofascial pain [40], and TMD pain [41], joined, in this review, the broad category of “TMD diagnosis.” In other words, they gather a generic “TMD diagnosis” entity appropriate for epidemiologic studies—in opposition to old-fashioned approaches, assessing punctual temporomandibular signs and symptoms, grouped into distinct “signs/symptoms” category for purposes of this review [45, 57].

Several instruments are available to assess stress and anxiety in the research environment [58], like the “Perceived Stress Scale” [59], the “State-Trait Anxiety Inventory for Adults” (STAI-AD) [60], and the “Stress and Adversity Inventory for Adults” (Adult STRAIN) [61]. For this review purpose, which focuses on labor stress and TMD, only original studies employing questionnaires targeting stress/occupational stress or anxiety combined with stress in the same instrument were considered. As discussed before, the term “distress” was accepted and included as a corresponding of stress [27, 28]. Conceptual and methodological issues regarding work stress evaluation in its numerous aspects and TMD are anything but simple. The lack of valid and reliable diagnostic tools for distinguishing work stress from a generic concept of stress (“day life” stress) and the fragmented work stress approach seem to represent an additional critical point in many of the selected articles. They possibly account for part of the significant heterogeneity. For example, individual relevant factors related to work stress, like work team relationships and workload, were sometimes not associated with TMD [48].

The quality of the evidence, both from Joanna Briggs Institute and GRADE, was low. All studies had problems in at least one Joanna Briggs tool domain. The issues included lack of confounder adjustment, valid instruments, cross-sectional study designs, the indirectness derived from the wide range of work categories assessed, and lack of a specific work stress assessment instrument accounted for it. On the other hand, it is essential to point out that RCT is not feasible, and only observational studies can be conducted. Hence, the low GRADE is not necessarily a fault of the researchers of the primary studies.

Stress is connected with systemic severe and potentially fatal diseases [62–64]. Human work activity is also cited as a potential source of stress, increasing medical disease risks [18, 65]. Both work stress [66] and TMD [67] affect the quality of life. Although TMD is a condition highly connected with the generic stress (daily life stress) in the literature [12–14], work stress and TMD are not traditionally investigated, unlike other musculoskeletal disorders in the workplace [68, 69]. Hence, future research efforts in the temporomandibular area should be directed to particular stress characteristics or domains, like occupational stress.

### 4.1. Strengths and Limitations

This systematic review is one of few (if not unique) to deal with work stress and TMD. Other relevant aspects are the distinction between TMD diagnostic and TMD signs and symptoms, apart from distinguishing valid from nonvalid TMD or stress assessment tools. Still, we used the Joanna Briggs Institute's tools for cross-sectional studies and the GRADE system to analyze methodological quality and the evidence's certainty, respectively. We searched in several databases, gray literature, and hand searched the included studies. However, publication bias is suspected for observational studies as registries in electronic databases are not mandatory [52].

The applicability to all work categories is limited due to limited professional classes included, which is considered indirectness. The heterogeneity was high for methodological aspects like the work category assessed, definitions of stress and TMD and assessment instruments, presence or categorization of control groups, scales' cut-off points, and statistical tests. For this reason, the evidence is narratively described together with the certainty of the evidence instead of pairwise meta-analysis comparing exposure and comparison groups.

### 4.2. Implications for Practice and Research

In the future, we expect more eligible epidemiologic studies with sound methods for selecting the appropriate stress-linked work categories [65], adequate control groups, sufficient confounder adjustment in statistical analysis, and valid and reliable diagnostic tools for both work stress and TMD. Such enhancement can provide more robust and stratified outcomes for impacting both clinical decisions and public health.

## 5. Conclusion

With high methodological discrepancies concerning diagnostic standards, sample characteristics, and control group criteria, there is a very low certainty of the association between work stress and TMD, so their relationship remains inconclusive by the available data.

## Figures and Tables

**Table 1:** Main descriptive features of selected studies (*n* = 12).

Author, year, country	Sample size (final)	Group 1 (*n*/%)	Group 2 (*n*/%)	Group 3 (*n*/%)	Group 4 (*n*/%)	Group 5 (*n*/%)	Stress: diagnostic tools, validation	TMD: diagnostic tools, validation	Results	Conclusion
Rantala et al., 2003, Finland	1339	Employees of a Finnish Broadcasting Company with low perceived stress (1020/76%)	Employees of a Finnish Broadcasting Company with high perceived stress (316/24%)				Occupational Stress Questionnaire (OSQ); it is not possible to check the validity of the stress scale.	TMD painless symptoms scale; it was not possible to evaluate the diagnosis validity.	High perceived stress group (50 TMJ-related painless symptoms out of 316) Low perceived stress group (75 TMJ-related painless symptoms out of 1020) (chi-square test; *p* < 0.001)	There was an association between stress and TMJ symptoms.
Nishiyama et al., 2012, Japan	2203	Electronic industry workers without TMD (1841/84%)	Electronic industry workers with TMD (362/16%)				Items 5–8 for psychosocial factors, including stress; variable is not validated.	Four-item questionnaire screening for patients with TMD-related symptoms (TRS); validated diagnosis.	Stress level and TMD were not associated after logistic regression analysis. No odds ratio was presented for the association between stress level and TMD.	There was no association between stress level and TMD.
Emodi Perelman et al., 2015, Israel	140	General occupation group (48/34%)	Dentists (44/32%)	High-tech workers (48/34%)			Self-reported stress at work; variable is not validated.	Full axis I exam and diagnosis according to the RDC/TMD for myofascial pain; validated diagnosis.	Higher stress at work (chi-square test; *p* = 0.03) and myofascial pain (chi-square test; *p* = 0.02) for the high-tech and dentist groups compared with the general occupational group.	High-tech workers and dentists were more prone to have stress and TMD.
Saruhanoğlu et al., 2016, Turkey	124	Workers from call centers with low stress (14/11%)	Workers from call centers with medium stress (33/27%)	Workers from call centers with high stress (77/62%)			The stress level of the job; variable is not validated.	Questionnaire from the RDC/TMD, axis 2. The diagnosis is validated.	Frequency of gradual mouth opening (chi-square test; *p* = 0.651), TMJ pain (chi-square test; *p* = 0.312), and TMJ noise (chi-square test; *p* = 0.944) was similar between the stress group levels (chi-square tests).	There was no relation between TMD signs and symptoms and stress in call center employees.
Martins et al., 2016, Brazil	104	Industrial workers with less stress (98/94%)	Industrial workers with more stress (6/6%)				Social Readjustment Rating Scale (SRRS); variable is validated.	Fonseca Anamnesis Index; outcome is validated.	There were 34 workers with TMD out of 98 with less stress and 3 workers with TMD among 6 with high stress levels (Fisher exact test, *p* = 0.663).	There was no association between stress and TMD.
Amorim and Jorge et al., 2016, Portugal	93	Violinists least anxious/stressed (46/49%)	Violinists most anxious/stressed (47/51%)				Kenny Music Performance Anxiety Inventory for anxiety and psychological distress; variable is validated.	Fonseca Anamnestic Questionnaire; the outcome is validated.	Music performance anxiety was associated with TMD scores (OR = 6.03; 95% CI 2.51–15.33) in the final logistic regression model.	Anxiety and distress were associated with TMD.
Amalina et al., 2018, Indonesia	92	Nurses without TMD (37/40%)	Nurses with TMD (55/60%)				Expanded Nursing Stress Scale (ENSS); variable is validated.	ID-TMD questionnaire, from RDC/TMD; the outcome is validated.	There was no association between TMD and the scores of ENSS: death and dying (Mann–Whitney *U* test; *p* = 0.177); conflict with physicians (independent *t*-test; *p* = 0.155); inadequate preparation (Mann–Whitney *U* test; *p* = 0.521); problems with peers (Mann–Whitney *U* test; *p* = 0.377); problems with supervisors (independent *t*-test; *p* = 0.107); workload (independent *t*-test; *p* = 0.091).	TMD was not associated with work stress among nurses in a type C Indonesian private hospital.
Gayathri et al., 2018, India	153	Software companies and IT professionals without stress (46/30%)	Software companies and IT professionals with stress (107/70%)				A self-administered online questionnaire for general stress symptoms; variable is not validated.	Self-admin. online questionnaire for TMD signs/symptom; outcome is not validated.	Stress level and TMD (Pearson's chi-square test; *p* < 0.005); there was no information on the frequencies of TMD between the groups.	There was an association between stress and TMD.
Han et al., 2018, South Korea	1612	Full-time female workers with low stress (1049/65%)	Full-time female workers with high stress (563/35%)				Self-reported stress; the variable is not validated.	TMD screening questions according to American Academy of Orofacial Pain (AAOP) and RDC/TMD; the outcome is validated.	There were 108 workers with TMD out of 1049 with less stress and 99 workers with TMD among 563 with high stress levels (chi-square test, *p* < 0.001).	There was an association between high stress and TMD among female workers.
Van Selms et al., 2019, Netherlands	515	Amateur/semiprofessional musicians for whom loading of the masticatory system is not required (209/40%)	Amateur/semiprofessional vocalists (306/60%)				A single question about the overall amount of stress experienced during the last 30 days (NRS 0-10); variable is not validated.	Symptom Questionnaire (SQ) of the DC/TMD; validated diagnosis.	No association in the final multiple regression model for both TMD pain and TMJ sounds. No odds ratios were presented for the association between stress level and TMD and TMJ sounds.	Stress level was not associated with both TMD pain and TMJ sounds.
Tay et al., 2019, Singapore	2043	Asian military personnel without TMD (1301/64%)	Asian military personnel with TMD (742/36%)				Stress subscale of DASS-21; variable is validated.	Symptom Questionnaire (SQ) of the DC/TMD; outcome is validated.	The mean values of DASS-21 stress subscale scores were 1.95 (SD = 2.85) and 3.29 (SD = 3.82) among those without and with TMD, respectively (Mann–Whitney *U* test; *p* = 0.001).	There was an association between stress and TMD.
Van Selms et al., 2020, Netherlands	1461	Control: other instrumentalists (208/15%)	Woodwind (371/25%)	Brass (300/20%)	Upper strings instrumentalists (276/19%)	Vocalists (306/21%)	Single question: “how much stress did you experience in daily life during the last 30 days?” Variable is not validated.	Symptom Questionnaire (SQ) of DC/TMD; the outcome is validated.	No association in the final multiple regression model for TMD pain. There is an association between TMJ sounds and stress (OR 1.09; 95% CI 1.02-1.16; *p* = 0.009).	There was an association between TMJ sounds and performance stress.

**Table 2:** The analysis of certainty of the evidence. Imported from GRADEpro Guideline Development Tool (GDT) (https://gdt.gradepro.org/app/#projects).

Certainty assessment							Impact	Certainty	Importance
No. of studies	Study design	Risk of bias	Inconsistency	Indirectness	Imprecision	Other considerations			
12	Observational studies	Very serious^a^	Very serious^b^	Very serious^c^	Not serious	Publication bias strongly suspected^d^	Seven studies found an association between stress and DTM or TMD signs and symptoms, and five studies found no association.	⨁◯◯◯ Very low	

CI: confidence interval. ^a^Twelve studies did not adjust for the confounders. Observational studies are at risk of bias because of differences in prognosis in exposed and unexposed populations (Guyatt et al., 2011//guidelines 4). ^b^There was great heterogeneity of instruments used for stress and TMD: 7 out of 12 studies used nonvalidated scales for stress (Nishiyama et al., 2012; Perelman et al., 2015; Saruhanoğlu et al., 2016; Han et al., 2018; M G et al., 2018; van Selms et al., 2019; van Selms et al., 2020) and 2 for TMD (Rantala et al., 2003; M G et al., 2018). Overall, only 3 articles employed valid instruments for both stress and TMD (Tay et al., 2019; Amorim and Jorge, 2016; Amalina et al., 2018). There was inconsistency among study findings: seven of selected articles found an association between TMD or TMD signs and symptoms and stress (Rantala et al., 2003; Perelman et al., 2015; Amorim and Jorge, 2016; Han et al., 2018; M G et al., 2018; Tay et al., 2019; van Selms et al., 2020), and 5 did not find an association (Nishiyama et al., 2012; Martins et al., 2016; Amalina et al., 2018; van Selms et al., 2019; van Selms et al., 2020). ^c^The evidence is from some types of categories of workers, with limited applicability to all workers. The majority of the evidence is from studies that evaluated stress in general, but not work stress (considered in only two studies: Amorim and Jorge (2016) and Amalina et al. (2018)). ^d^Observational studies are more prone to publication bias than RCTs or clinical trials due to the nonmandatory registration in databases (Guyatt et al., 2011/guidelines 5).

## Data Availability

The data used to support the findings of this study are available from the corresponding author upon request.

## Supplementary Materials

Supplementary 1 Search strategies according to electronic databases (inception May 2020).

Supplementary 2 Link for supplementary file 2 https://onedrive.live.com/view.aspx?resid=E2CC007F59012677!1469&ithint=file%2cxlsx&authkey=!AGN-amaFs_6OLSQ.

Supplementary 3 The assessment of methodological quality with the Joanna Briggs Institute (JBI) tool for cross-sectional studies.
