# Supplementary material for: Association between Stress at Work and Temporomandibular Disorders: A Systematic Review
Source: Biomed Res Int. 2021 May 15;2021:2055513. doi: 10.1155/2021/2055513 (PMC8249225; doi:10.1155/2021/2055513)
Supplement: Supplementary 1 — Search strategies according to electronic databases (inception May 2020). [file 2055513.f1.docx]

| **SF 1.** Search strategies according to electronic databases (inception May 2020) | |
| --- | --- |
|  | **PubMed** |
| #1 | (social workers OR health personnel OR occupational groups OR government employees OR employment OR income OR remuneration OR salaries OR caregivers OR nurses OR dentists OR physicians OR military personnel OR educational personnel OR school teachers OR singing OR singer OR police OR nursing assistants OR public sector OR private sector OR industry OR commerce OR economics OR personnel management OR staff development OR auxiliaries OR worker OR employees OR labor force OR musician OR music player) |
| #2 | **AND** (occupational stress OR Burnout, professional OR occupational diseases OR stress, psychological OR job stress OR work-related stress OR stress, workplace OR professional stress OR occupational health OR employee health OR Unemployment OR workload OR shift work schedule OR occupational exposure) |
| #3 | **AND** (temporomandibular joint disorders OR temporomandibular joint dysfunction syndrome OR disorder, TMJ OR disorder temporomandibular OR disease, temporomandibular joint OR myofascial pain syndromes) |
| #4 | **AND** (cross-sectional studies OR epidemiologic studies OR studies, prevalence OR observational study OR case-control studies OR retrospective studies OR cohort studies OR longitudinal studies OR prospective studies OR incidence studies) |

|  | **Lilacs** |
| --- | --- |
| #1 | (“Pessoal de Saúde” OR “Categorias de Trabalhadores” OR “Empregados do Governo” OR “labor force” OR emprego OR workers OR nurses OR dentists OR physicians OR “military personnel” OR “educational personnel” OR “school teachers” OR polícia OR “public sector” OR “private sector” OR industry OR commerce OR economics) |
| #2 | **AND** (“Estresse Ocupacional” OR “Burnout, professional” OR “Esgotamento Profissional” OR “Estresse Psicológico” OR “occupational health” OR workload OR “occupational exposure” OR “professional stress” OR “work-related stress” OR “job stress” OR saúde do trabalhador OR “occupational stress” OR “work stress”) |
| #3 | **AND** (“Transtornos da Articulação Temporomandibular” OR “Síndrome da Disfunção da Articulação Temporomandibular” OR “temporomandibular joint dysfunction syndrome” OR “temporomandibular joint disorders”) |

|  | **Embase** |
| --- | --- |
| #1 | ('social worker'/exp OR 'health care personnel'/exp OR 'named groups by occupation'/exp OR 'occupational groups' OR 'government employee'/exp OR 'employment'/exp OR 'income'/exp OR 'remuneration'/exp OR 'salary and fringe benefit'/exp OR 'caregiver'/exp OR 'nurse'/exp OR 'dentist'/exp OR 'physician'/exp OR 'military personnel'/exp OR 'teacher'/exp OR 'singer'/exp OR 'police'/exp OR 'nursing assistant'/exp OR 'public sector'/exp OR 'private sector'/exp OR 'industry'/exp OR commerce OR 'economics'/exp OR 'personnel management'/exp OR 'staff development' OR 'auxiliaries' OR 'worker'/exp OR 'employees' OR 'workforce'/exp) |
| #2 | **AND** ('job stress'/exp OR 'professional burnout'/exp OR 'occupational disease'/exp OR 'mental stress'/exp OR 'occupational stress' OR 'professional stress' OR 'occupational health'/exp OR 'unemployment'/exp OR 'workload'/exp OR 'shift schedule'/exp OR 'occupational exposure'/exp) |
| #3 | **AND** ('temporomandibular joint disorder'/exp OR 'temporomandibular joint dysfunction syndrome' OR 'disorder, TMJ' OR 'myofascial pain'/exp) |
| #4 | **AND** ('cross-sectional study'/exp OR 'epidemiologic studies' OR 'prevalence'/exp OR 'observational study'/exp OR 'case control study'/exp OR 'retrospective study'/exp OR 'cohort analysis'/exp OR 'cohort studies' OR 'longitudinal study'/exp OR 'prospective study'/exp OR 'incidence'/exp) |

|  | **Scopus** |
| --- | --- |
| #1 | (“social worker” OR “health care personnel” OR “occupational groups” OR “government employee” OR “employment” OR “income” OR “remuneration” OR “salary and fringe benefit” OR caregiver OR nurse OR dentist OR physician OR “military personnel” OR teacher OR singer OR police OR “nursing assistant” OR “public sector” OR “private sector” OR industry OR commerce OR economics OR “personnel management” OR “staff development” OR auxiliaries OR worker OR employees OR workforce OR musician OR music player) |
| #2 | **AND** (“job stress” OR “professional burnout” OR “occupational disease” OR “mental stress” OR “occupational stress” OR “professional stress” OR “occupational health” OR unemployment OR workload OR “shift schedule” OR “occupational exposure”) |
| #3 | **AND** (“temporomandibular joint disorder” OR “temporomandibular joint dysfunction syndrome” OR “disorder, TMJ” OR “myofascial pain”) |
| #4 | **AND** (“cross-sectional study” OR “epidemiologic studies” OR prevalence OR “observational study” OR “case control study” OR “retrospective study” OR “cohort studies” OR “longitudinal study” OR “prospective study” OR incidence) |

|  | **Web of Science** |
| --- | --- |
| #1 | TS=(social workers OR health personnel OR occupational groups OR government employees OR employment OR income OR remuneration OR salaries OR caregivers OR nurses OR dentists OR physicians OR military personnel OR educational personnel OR school teachers OR singing OR singer OR police OR nursing assistants OR public sector OR private sector OR industry OR commerce OR economics OR personnel management OR staff development OR auxiliaries OR worker OR employees OR labor force) |
| #2 | TS=(occupational stress OR Burnout, professional OR occupational diseases OR stress, psychological OR job stress OR work-related stress OR stress, workplace OR professional stress OR occupational health OR employee health OR Unemployment OR workload OR shift work schedule OR occupational exposure) |
| #3 | TS=(temporomandibular joint disorders OR temporomandibular joint dysfunction syndrome OR disorder, TMJ OR disorder temporomandibular OR disease, temporomandibular joint OR myofascial pain syndromes |
| #4 | TS=(cross-sectional studies OR epidemiologic studies OR studies, prevalence OR observational study OR case-control studies OR retrospective studies OR cohort studies OR longitudinal studies OR prospective studies OR incidence studies) |
| #5 | #1 AND #2 AND #3 AND #4 |

|  | |
| --- | --- |
|  | |
|  | **OpenGrey and Google Scholar** |
| #1 | (social workers OR health personnel OR occupational groups OR government employees OR employment OR income OR remuneration OR salaries OR caregivers OR nurses OR dentists OR physicians OR military personnel OR educational personnel OR school teachers OR singing OR singer OR police OR nursing assistants OR public sector OR private sector OR industry OR commerce OR economics OR personnel management OR staff development OR auxiliaries OR worker OR employees OR labor force OR musician OR music player) |
| #2 | **AND** (occupational stress OR Burnout, professional OR occupational diseases OR stress, psychological OR job stress OR work-related stress OR stress, workplace OR professional stress OR occupational health OR employee health OR Unemployment OR workload OR shift work schedule OR occupational exposure) |
| #3 | **AND** (temporomandibular joint disorders OR temporomandibular joint dysfunction syndrome OR disorder, TMJ OR disorder temporomandibular OR disease, temporomandibular joint OR myofascial pain syndromes) |
| #4 | **AND** (cross-sectional studies OR epidemiologic studies OR studies, prevalence OR observational study OR case-control studies OR retrospective studies OR cohort studies OR longitudinal studies OR prospective studies OR incidence studies) |
