# Supplementary material for: Association between Stress at Work and Temporomandibular Disorders: A Systematic Review
Source: Biomed Res Int. 2021 May 15;2021:2055513. doi: 10.1155/2021/2055513 (PMC8249225; doi:10.1155/2021/2055513)
Supplement: Supplementary 2 — Link for supplementary file 2 https://onedrive.live.com/view.aspx?resid=E2CC007F59012677!1469&ithint=file%2cxlsx&authkey=!AGN-amaFs_6OLSQ. [file 2055513.f2.docx]

Association between stress at work and temporomandibular disorders: a systematic review

Link for Supplementary file 2

https://onedrive.live.com/view.aspx?resid=E2CC007F59012677!1469&ithint=file%2cxlsx&authkey=!AGN-amaFs_6OLSQ
