# Supplementary material for: Association between Stress at Work and Temporomandibular Disorders: A Systematic Review
Source: Biomed Res Int. 2021 May 15;2021:2055513. doi: 10.1155/2021/2055513 (PMC8249225; doi:10.1155/2021/2055513)
Supplement: Supplementary 3 — The assessment of methodological quality with the Joanna Briggs Institute (JBI) tool for cross-sectional studies. [file 2055513.f3.docx]

| Authors | Were the criteria for inclusion in the sample clearly defined? | Were the study subjects and the setting described in detail? | Was the exposure measured in a valid and reliable way? | Were objective, standard criteria used for measurement of the condition? | Were  confounding factors identified? | Were strategies to deal with confounding factors stated? | Were the outcomes measured in a valid and reliable way? | Was appropriate statistical analysis used? |
| --- | --- | --- | --- | --- | --- | --- | --- | --- |
| Rantala et al., 2003 | No | Yes | Unclear | Yes | No | No | No | Yes |
| Nishiyama et al., 2012 | No | Yes | No | Yes | No | No | Yes | Yes |
| Perelman et al., 2015 | Yes | Yes | No | Yes | No | No | Yes | No |
| Amorim & Jorge, 2016 | Yes | Yes | Yes | Yes | No | No | Yes | Yes |
| Saruhanoğlu et al., 2016 | Yes | Yes | No | Yes | No | No | Yes | Yes |
| Martins et al., 2016 | No | No | Yes | Yes | No | No | Yes | Yes |
| Han et al., 2018 | No | Yes | No | Yes | No | No | Yes | Yes |
| Gayathri et al., 2018 | Yes | Yes | No | Yes | No | No | No | No |
| Amalina & Tanti et al., 2018 | Yes | Yes | Yes | Yes | No | No | Yes | Yes |
| Van Selms et al., 2019 | Yes | Yes | No | Yes | No | No | Yes | Yes |
| Tay et al., 2019 | Yes | Yes | Yes | Yes | No | No | Yes | Yes |
| Van Selms et al., 2020 | Yes | Yes | No | Yes | No | No | Yes | Yes |
